# Factors Influencing the Answerability and Popularity of a Health-Related Post in the Question-and-Answer Community: Infodemiology Study of Metafilter

**DOI:** 10.2196/48858

**Published:** 2023-11-17

**Authors:** Jinqing Yang, Zhifeng Liu, Qicong Wang, Na Lu

**Affiliations:** 1 School of Information Management Central China Normal University Wuhan China; 2 Department of Information Management Peking University Beijing China; 3 School of Economics and Management Hainan Normal University Haikou China

**Keywords:** user behavior, dynamic network analysis, health consultation, health question and answers community, question-and-answer, Q&A, negative binomial regression

## Abstract

**Background:**

The web-based health question-and-answer (Q&A) community has become the primary and handy way for people to access health information and knowledge directly.

**Objective:**

The objective of our study is to investigate how content-related, context-related, and user-related variables influence the answerability and popularity of health-related posts based on a user-dynamic, social network, and topic-dynamic semantic network, respectively.

**Methods:**

Full-scale data on health consultations were acquired from the *Metafilter* Q&A community. These variables were designed in terms of context, content, and contributors. Negative binomial regression models were used to examine the influence of these variables on the favorite and comment counts of a health-related post.

**Results:**

A total of 18,099 post records were collected from a well-known Q&A community. The findings of this study include the following. Content-related variables have a strong impact on both the answerability and popularity of posts. Notably, sentiment values were positively related to favorite counts and negatively associated with comment counts. User-related variables significantly affected the answerability and popularity of posts. Specifically, participation intensity was positively related to comment count and negatively associated with favorite count. Sociability breadth only had a significant impact on comment count. Context-related variables have a more substantial influence on the popularity of posts than on their answerability. The *topic diversity* variable exhibits an inverse correlation with the comment count while manifesting a positive correlation with the favorite count. Nevertheless, *topic intensity* has a significant effect only on favorite count.

**Conclusions:**

The research results not only reveal the factors influencing the answerability and popularity of health-related posts, which can help them obtain high-quality answers more efficiently, but also provide a theoretical basis for platform operators to enhance user engagement within health Q&A communities.

## Introduction

### Background

In the era of digital information, a considerable amount of information is available on the internet, with health and lifestyle information drawing significant attention [1]. In recent years, a growing number of people have turned to the internet to search for health-related information, particularly since the outbreak of the COVID-19 pandemic. The public urgently needs health-related information on how to improve their well-being [2]. With advanced Web 2.0 technologies, the general public can now easily produce, share, and access health-related information on the internet. People can access health-related information through search engines or ask for answers through online health question-and-answer platforms (OHQPs). OHQPs are attracting more attention from users because the public can directly post health-related questions to access the required health information [3]. Asking questions on OHQPs is the most straightforward and convenient way for users to address health concerns. Specifically, people pose health-related questions to seek answers and valuable information from OHQPs, which is the process of health consultations. However, an assortment of internal and external factors, such as information literacy, expression ability, and questioning topics, may modulate the caliber and efficacy of user health consultations.

The usefulness and value of user posts are directly displayed in questions that are inclined to be answered and favored. The number of answers and favorites could directly react to the effectiveness of user consultation [4,5]. Thus, exploring the internal and external factors that impact the effect of user consultation could contribute to improving consultation behavior and obtaining the most relevant health information to meet needs. In OHQPs, users mostly consult on their requirements by posting a question. More specifically, internal factors primarily involve user-related aspects, such as sociability and participation. External factors, in contrast, are related to the content and context of posts, such as readability, topic diversity, and emotional tone. Although some studies have explored the answerability and popularity of posts in question-and-answer (Q&A) communities [6-8], few researchers have specifically explored health consultation concerns based on comprehensive dynamic behavior data in the Q&A community. Certain variables, such as user sociability and the topic prevalence of posts, are dynamic and change over time.

To address the above issues, this study gathers all activity records of users from the *Metafilter* Q&A community and constructs a user-dynamic social network and topic-dynamic semantic network. Negative binomial and Poisson regression models were used to examine the effects of internal and external factors on answerability and popularity, respectively. The findings of this study not only enable users to pose high-quality questions in the context of their health consultation demand to obtain valuable answers quickly but also assist Q&A administrators to manage their platforms more effectively and scientifically.

### Literature Review and Hypotheses

Q&A communities have emerged as vital platforms for users to exchange health-related knowledge [8]. In these communities, knowledge sharing primarily relies on users posing questions and their peers providing answers [9]. Consequently, it is crucial to understand the factors that impact the answerability and popularity of posts to boost user engagement and enhance the overall quality of Q&A platforms. Numerous studies have investigated the factors influencing the answerability and popularity of posts within Q&A communities.

The answerability of posts is a pressing concern, as many posts remain unanswered in Q&A communities, leaving users’ needs unmet [10]. To tackle the growing number of unanswered posts, some studies are investigating the factors influencing post answerability. For example, Maity et al [11] discovered that a post’s linguistic structure significantly affects its answerability. Currently, other studies have found that the level of detail, specificity, clarity, and emotional tone of a post’s content also substantially affects its answerability [6]. In addition, some studies have explored using variables about the posts’ text, such as the title and content, to predict the number of comments a post might receive [12].

Moreover, the answerability of a post can be influenced by the popularity and engagement level of the user consulting the question, as well as the timing of the post’s submission [11]. One study used the *XGBoost* machine learning algorithm to predict whether questions on Stack Overflow would receive answers and found that factors such as the timing of the post and its tags played a significant role [13]. Other researchers have discovered that the topic of the post, the experience of user consulting, and the readability of the post text significantly influence whether Stack Overflow questions are answered [14].

In contrast, *the popularity of posts* in a Q&A community is also a crucial aspect to consider as it plays a vital role in the community’s development. When a post is more popular, it fosters the exchange and dissemination of knowledge [15]. Li et al [5] discovered that factors such as the length, vividness, and topic of health-related post text, as well as the gender and education level of the users, significantly impact the popularity of posts. Furthermore, Liu et al [16] investigated how content-related variables, social behavior, and user characteristics affect a post’s popularity. They also used supervised machine learning algorithms to predict post popularity. Similarly, research has demonstrated that factors such as post length and vividness, expertise and degree of centrality of contributors, and social interactions among members are significantly correlated with post popularity [17]. In addition, some studies have examined how social and content-related signals influence response behavior toward posts in Q&A communities for older adults [18].

**Figure 1:**
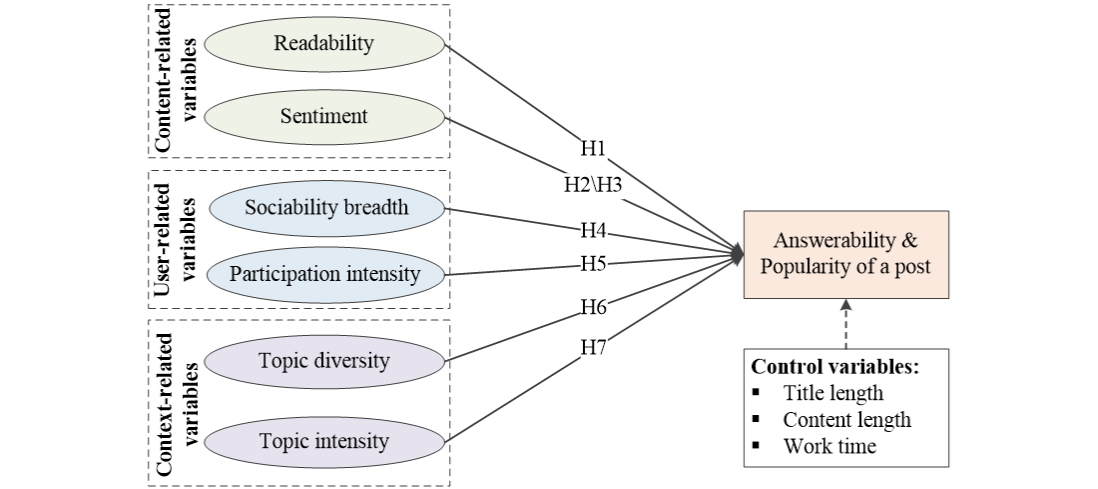
The research model of our study. H: Hypothesis.

According to previous studies, the factors influencing post answerability and popularity can be broadly divided into 3 main categories: content, context, and users. These categories play a critical role in determining the appeal and accessibility of posts on various web-based platforms. As a result, we propose hypotheses to explore the factors influencing the answerability and popularity of posts through the lenses of content, context, and user, with the ultimate goal of enhancing knowledge-sharing efficiency in web-based Q&A communities. Figure 1 illustrates the research model.

### Content-Related Variables

In our study, content-related variables encompassed the readability of the post and the emotional sentiment of the post. To some extent, the readability of a post reflects the difficulty in understanding the post text. If respondents can easily read the text of posts, they can better answer the post while saving time. Previous research has also shown that the readability of a post text is significantly related to its answerability [19]. In general, the title of a post is much shorter relative to its content. Therefore, the content readability of health-related posts may be related more to their answerability and popularity. Thus, we propose the following research hypothesis:

- H 1: The readability of a post’s content will be related to its answerability and popularity.

In addition to readability, existing literature suggests that the sentiment of a post text may be related to its answerability and popularity [20]. If a post exhibits a more evident emotional inclination, it is generally more likely to evoke emotional resonance and attention from people, leading to more intense discussions surrounding the post and ultimately resulting in higher answerability and popularity. Hence, we propose the following hypotheses:

- H 2: The sentiment of a post’s title will be related to its answerability and popularity.
- H 3: The sentiment of a post’s content will be related to its answerability and popularity.

### User-Related Variables

The answerability and popularity of a post are not solely dependent on its content, but also on the characteristics of users. Specifically, users’ position within the community, such as their centrality, plays a crucial role in determining the response and interest in their inquiries. Centrality of users refers to their degree of centrality in the community’s social network, indicating their status within the community. Research indicates that users are more inclined to reply to posts from knowledge seekers with higher job ranks in Q&A communities [7]. In this study, we constructed 2 distinct networks based on interconnections and author interactions in the context of question exchanges: the following relationship network and Q&A relationship network. The following relationship was created when one followed another, and the Q&A relationship was established when one comments on a post of one another. The user’s centrality regularly measures their importance in a social network [21,22]. Moreover, the degree of centrality represents the user’s *sociability breadth* in the following relationship network to measure their followings from other users. Furthermore, the degree centrality signifies *participation intensity* in the Q&A relationship network to measure participation in answering the questions. This methodology facilitated a comprehensive examination of the relationships and interactions among community members, enabling an analysis of how these connections may impact the popularity and answerability of posts within the community.

According to social capital theory [23], if a user has a higher network centrality, it means that they have established replies with more community members and possess greater social capital. Social capital is beneficial in obtaining better benefits within a network. Moreover, prior research has partially confirmed that a positive correlation exists between the degree centrality of a user and the number of responses that the user receives [17]. Hence, the higher the degree centrality of the user in the Q&A relationship network and the following relationship network, the greater is the probability that the question asked may be answerable and popular. Thus, we postulate the following hypothesis.

- H 4: The sociability breadth of users will be related to the answerability and popularity of the posts.
- H 5: The participation intensity of users will be related to the answerability and popularity of the posts.

### Context-Related Variables

According to signal theory [24], information transmission is influenced not only by the sender and content of the message, but also by the environment. In this study, the answerability and popularity of posts may have been influenced by the topic context. Posts related to trending or popular topics were more likely to attract attention and generate engagement. Similarly, posts in an environment where the topic is highly relevant and timely are more likely to be answered by knowledgeable individuals, leading to increased popularity and visibility. Previous research has indicated that post tags have an impact on their popularity in the Stack Overflow community [4]. This study further explores the impact of posts’ topics on their answerability and popularity from the perspectives of topic diversity and intensity. Consequently, we posit the following hypothesis:

- H 6: Topic diversity will be related to the answerability and popularity of the posts.
- H 7: Topic intensity will be related to the answerability and popularity of the posts.

### Control Variables

With reference to previous studies, the control variables included work time and the length of posts (the posts’ title and content), all of which were considered. Work time refers to whether the user who asks the question is working hours. Previous research results have shown that the time of posing a question could affect the knowledge exchange and collaboration among respondents. For example, Adaji et al [25] found that questions posted in the evening scored higher than those posted during the day did. According to the daily working hours stipulated by the US Fair Labor Standards Act of 1938 [26], work hours are from 09:00 AM to 5:00 PM. In this study, the independent variable “question creation time” was divided into working hours (9 AM-5 PM), represented by 1, and nonworking hours (other times), represented by 0. According to this, we propose the following hypothesis:

- H 8: The timing of the questions asked by users, whether during working hours or not, will be related to its answerability and popularity.

The title and content length of posts can, to some extent, represent the amount of information contained. The longer the title and content of a post, the more information it contains. If community members can obtain sufficient information from the text of a post, it is more conducive to understanding the needs of users, and the poster is more likely to be answered [27]. Simultaneously, if the text of a post contains a higher amount of information, it may possess greater informational value and be more likely to be saved, leading to the increased popularity of the post. Thus, we propose the following hypothesis:

- H 9: The length of a post’s title will be related to its answerability and popularity.
- H 10: The length of a post’s content will be related to its answerability and popularity.

To summarize the above, detailed variable descriptions are presented in Table 1 to better calculate the hypothesis variables.

**Table 1:** The descriptions of the hypothesis variables.

Category and variables			Description
**Content-related variables**			
	Readability	Readability can be calculated by the *Flesch Reading-Ease* formula	
	Sentiment	Sentiment refers to, respectively indicate positive and negative emotions of the posts’ title and content	
**User-related variables**			
	Participation intensity	The user degree centrality in the Q&A^a^ relationship network	
	Sociability breadth	The user degree centrality in the following relationship network	
**Context-related variables**			
	Topic diversity	The tag count of a question that user posts	
	Topic intensity	The tag degree in the tag co-occurrence semantic network	
**Control variables**			
	Title length	Character length of a post’s title	
	Content length	Character length of a post’s content	
	Work time	Whether the user who asks the question is in working time	

^a^Q&A: question-and-answer.

## Methods

### Data Collection

The data set used in this study was obtained from the *Metafilter* database [28]. *MetaFilter* is a general-interest web-based community composed of *Metafilter*, *AskMeFi, FanFare, Projects, Music, Jobs, IRL*, and *MetaTalk* subsites. Detailed data situations are described in *data set description* of Multimedia Appendix 1.

### Data Calculation and Analysis

To verify the hypotheses given in this study, we need to perform calculations on some variables, such as text readability and sentiment. The dynamic user social network (ie, user Q&A relationship network and following relationship network) and tag semantic network can be used to calculate participation intensity, sociability, and topic intensity.

#### Text Readability Calculation

The reading-ease score of the post text can be calculated using the *Flesch Reading-Ease* formula [29]. Higher scores indicate that the post text is easier for users to read, whereas lower values imply that the post text is more challenging to comprehend. The formula for computing the *Flesch Reading-Ease Score* is as follows:



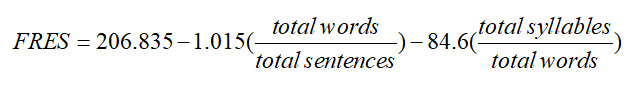



For example, Time magazine scores approximately 52, which means that the text is difficult to read.

#### Text Sentiment Analysis

Text sentiment trends can influence user behavior in Q&A communities [30]. Sentiment analysis is a common text-processing operation that often uses the *TextBlob* library to return the sentiment property. The sentiment scores were float values within the range (−1.0, 1.0). Positive and negative scores indicated positive and negative emotions, respectively.

#### Dynamic User Social Network Construction

As users participate in Q&A community activities, their reputations evolve continuously. A user social network consists of users and their associated relationships, such as Q&A interactions and followings. Specifically, when user A answers a question posted by user B, a Q&A relationship is established between users A and B. If user A follows user B, there is a link between them. The user’s social network can provide important variables for each post from the contributor’s perspective.

**Figure 2:**
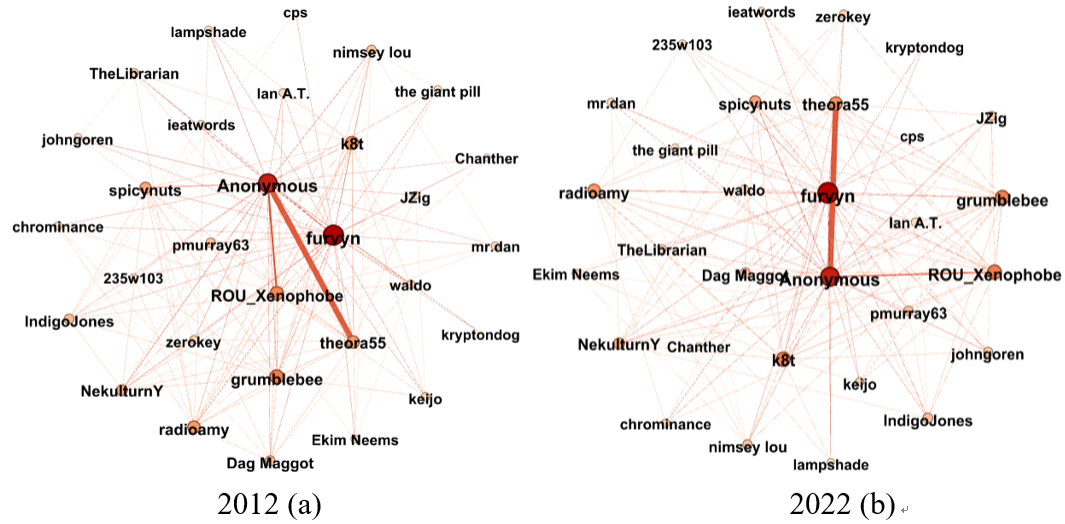
Dynamic egocentric question-and-answer network of user “furvyn.” (a) 2012; (b) 2022.

**Figure 3:**
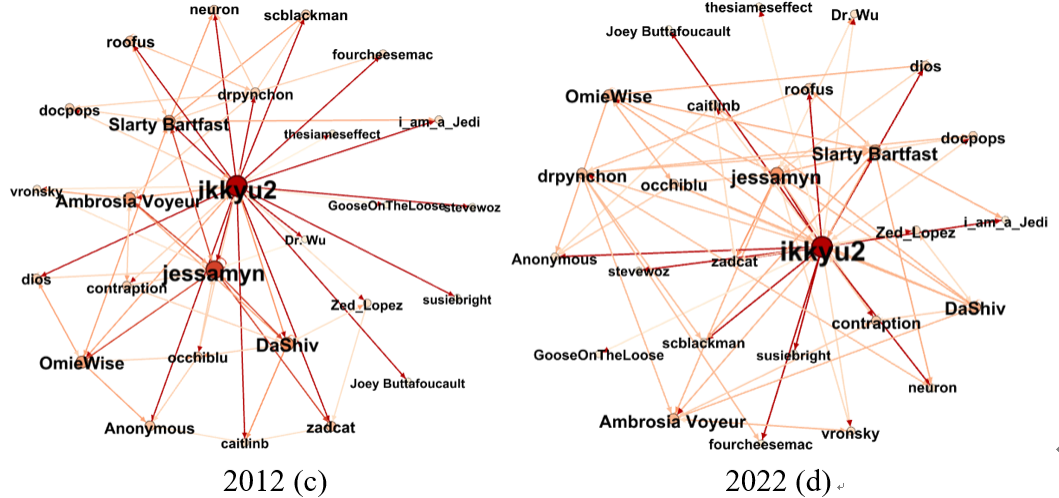
Dynamic egocentric following network of user “ikkyu2.” (c) 2012; (d) 2022.

Because the Q&A and the following relationships are established on the web in real time, the user social network is, by definition, a dynamic network. The dynamic user social network connected by Q&A and the following relationships are symbolized as 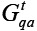 , and 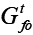, where 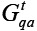 is an undirected network, and 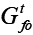 is a directed network. Post users’ centrality can be calculated from 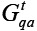 and 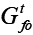 to represent sociability breadth and participation intensity. For a better insight, we extracted the egocentric networks of user “furvyn” and “ikkyu2,” respectively, from 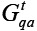 and 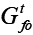, as shown in Figures 2 and 3.

#### Dynamic Tag Semantic Network Construction

**Figure 4:**
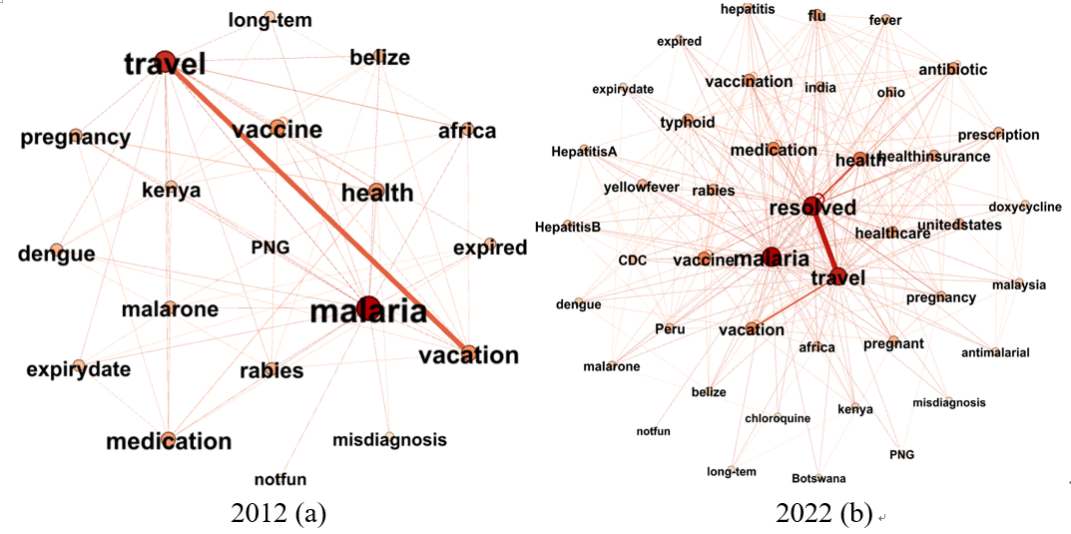
Dynamic egocentric semantic network of tag “malaria.” (a) 2012; (b) 2022.

Tags were added to express the semantic topics of a post. When a user creates or selects 2 tags to label a single post, a co-occurrence relationship is generated between them. A tag co-occurrence network can be referred to as a dynamic semantic network, denoted as 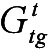. To minimize the effect of heteromorphic synonymous tags, word stems were extracted. The mean degree centrality can be calculated to represent post-topic intensity. *Malaria*, a life-threatening disease, was selected for the visualization. The egocentric network of tag “malaria” was extracted for visualization, as shown in Figure 4.

**Figure 5:**
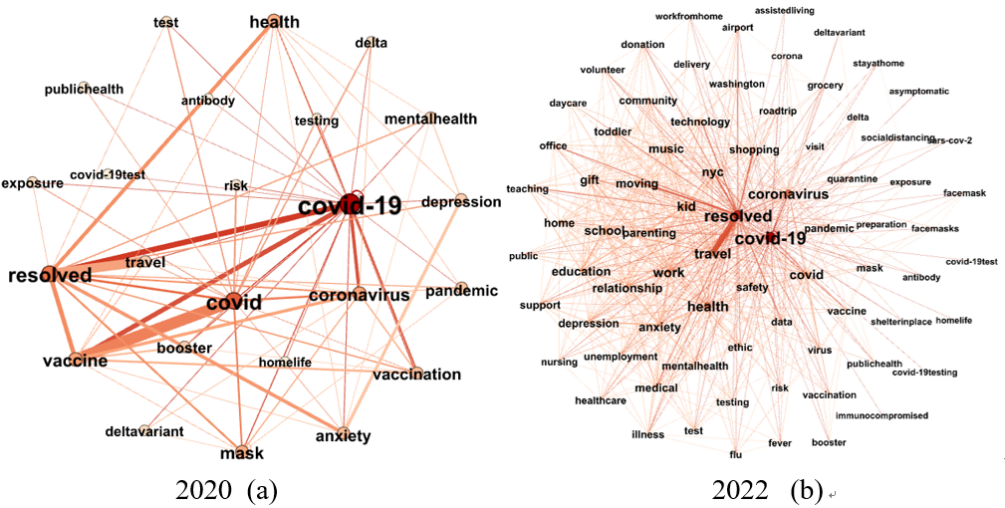
Dynamic egocentric semantic network of tag “COVID-19.” (a) 2020; (b) 2022.

To understand the topic intensity of the tags, we visualized the variation in centrality of COVID-19 in the dynamic semantic network in Figure 5.

**Figure 6:**
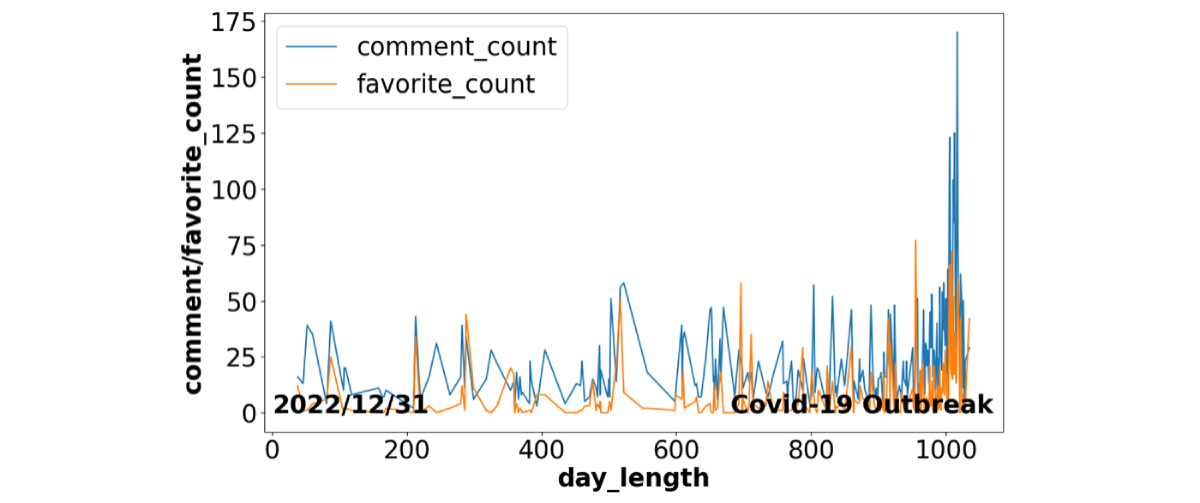
Time-series analysis about COVID-19 posts.

Observing Figure 5, it can be discerned that the proximal tags associated with “covid-19” predominantly concern emotional responses to the contagion, such as “anxiety,” “depression,” “mental health,” and “risk” during the initial dissemination phases of “covid-19.” By the year 2022, the primary focus shifts toward matters pertinent to daily life, including “shopping,” “travel,” “work,” “school,” etc. In addition, we conduct a time-series analysis pertaining to comment counts and favorite counts related to “covid-19” posts, as illustrated in Figure 6.

Figure 6 demonstrates that the COVID-19 outbreak received widespread attention early, and vigorous user communication declined as COVID-19 was gradually controlled.

In addition, a single post was labeled with more than 2 tags. The tag number can be used to measure the topic diversity. According to statistics, a post could have a maximum of 59 tags, a minimum of one tag, and a mean of 4.3 tags.

### Ethical Considerations

This study was approved by the Institutional Review Boards of the school of Information Management, Central China Normal University (CCNU-IRB-202310023b).

## Results

### Descriptive Statistics of the Variables

To comprehend the distribution of content-, user-, and context-related variables, we performed descriptive statistics for the variables used in the models. Specifically, we calculated the minimum, maximum, mean, SD, and variance inflation factors (VIFs), as shown in Table 2.

**Figure 7:**
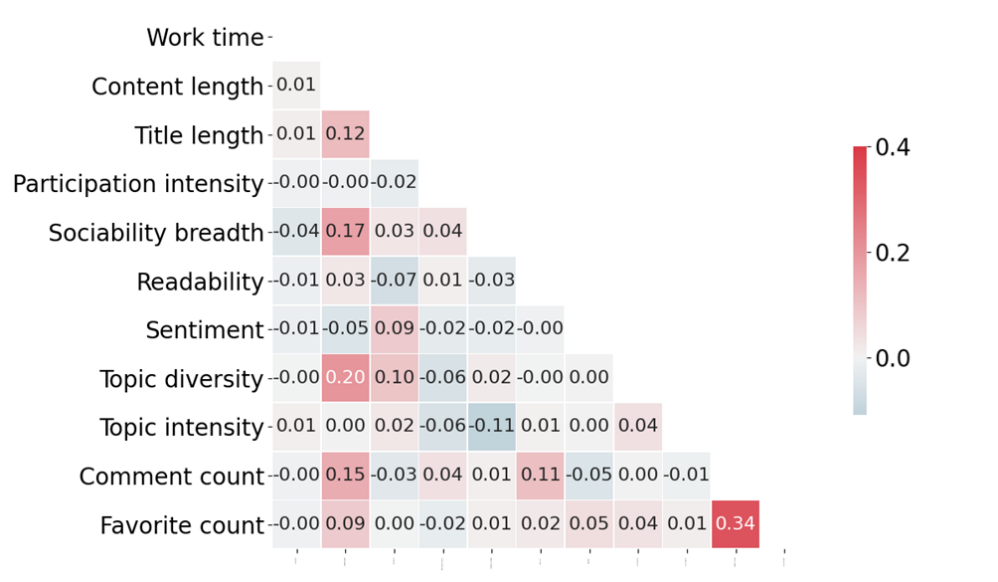
The correlation between the various variables.

Table 2 reports the descriptive statistics for the variables in our models. To corroborate the absence of substantial multicollinearity among the selected variables, we use the *VIF* to check the linear correlation between the variables. To ensure uniformity across all variables, the scaling technique—normalization is implemented for their standardization. The *VIF* values, computed using the variance_inflation_factor package, were consistently below 4, thereby indicating the nonexistence of multicollinearity within the regression models [31]. In addition, we illustrated the correlation coefficient matrix for the aforementioned independent and dependent variables, as shown in Figure 7.

Figure 7 demonstrates that a weak correlation exists between the various independent variables, with a maximum value of 0.20, and the correlation between the 2 dependent variables is 0.34.

**Table 2:** Results of descriptive statistics for the variables.

Category and variables			Values, mean (SD; range)		VIF^a^
**Content-related variables**					
	Content readability	71.09 (27.69; −640 to 121)		2.48	
	Title sentiment	0.03 (0.25; −1 to 1)		1.09	
	Content sentiment	0.06 (0.26; −1 to 1)		1.14	
**Contributor-related variables**					
	Participation intensity	30.2 (61.8; 0 to 345)		1.28	
	Sociability breadth	2437.4 (4753.05; 0 to 18,240)		1.04	
**Context-related variables**					
	Topic diversity	4.16 (2.20; 1 to 32)		2.74	
	Topic intensity	1658 (3709.6; 0 to 57,866)		1.21	
**Control variables**					
	Title length	40.6 (18.8; 1 to 242)		3.82	
	Content length	1325.6 (1091.8; 30 to 16,440)		2.64	
	Work time	0.48 (0.50; 0 to 1)		1.80	
**Dependent variables**					
	Comment count	14.9 (10.9; 0 to 137)		3.10	
	Favorite count	5.6 (11.8; 0 to 435)		1.41	

^a^VIF: variance inflation factor.

### Primary Results

The dependent variables (*ie, comment count and favorite count*) are count data, which are nonnegative integers. This experiment was analyzed using Poisson regression when the dependent variables were assumed to follow a normal distribution. However, after using the *Kolmogorov–Smirnov* test, we found that the significance coefficients of the 2 dependent variables were both less than 0.05, indicating that the normal distribution condition was not achieved. Therefore, the negative binomial regression model (*NBRM*) is suitable for estimating the theoretical hypotheses. According to Table 2, the independent variables were fed into the *NBRM* in the following order: (1) control variables, (2) content-related variables, (3) user-related variables, and (4) context-related variables.

The validity of the whole model was analyzed using the likelihood ratio test. Specifically, if the *P* value derived from the likelihood ratio test is less than .05, it indicates that the model is effective, whereas the opposite suggests that the model is not effective. The validity of the relevant variables for the *NBRM* was analyzed by comparing the changes in the *Akaike information criterion* before and after adding the given dependent variables. The independent variables were classified into 4 categories: control, content-related, contributor-related, and context-related. Thus, 4 models need to be performed for each dependent variable (comment count or favorite count). The likelihood ratio *P* values of the 4 models are all less .001, which means that all the models are effective. Specifically, Tables 3 and 4 show the effects of the 4 types on comment count (*answerability*) and favorite count (*popularity*) based on the results of the negative binomial regression model.

The answerability and popularity of posts are of utmost concern to health consultants in the Q&A community. Table 2 displays the factors influencing the responses based on content, context, and user-related variables. After incrementally feeding the 3 categories of variables, the Akaike Information Criterion values of the 2 NBRMs decreased based on the control variables, indicating that these variables were effective. However, different variables have distinct effects on the answerability and popularity of posts.

As for the control variables, the *P* values of the *Work time* variable are all higher than .05 in these models, indicating that both answerability and popularity are not influenced by the posting time. In Models 2 and 4, the *P* values of the *Content length and Title length* are smaller than .001, which proves Hypotheses *H9* and *H 10*. Moreover, the coefficients of the *Content length* and *Title length* variables are positive and negative, respectively, which indicates that users prefer posts with a short title.

In terms of content variables, the *P* value of content readability is smaller than .001 in *Models 2 and 4*, and the corresponding estimate values are positive and negative, respectively. The title and content are important components of the post text, and sentiment can be divided into title and content sentiments. The *P* values of title sentiment, content sentiment, and their absolute values are all smaller than .001 in *Models 2 and 4*. These results support hypotheses *H 1*, *H 2* and *H 3*. However, the estimated values of title and content sentiment are positively related to favorite count and negatively associated with comment count. This means that posts with negative emotions would receive more comments, and those with positive emotions would gain more favorites. It is worth noting that the coefficients of the absolute value of title and content sentiment (|content sentiment| and |title sentiment|) are both positive, which means that sentiment polarity has a significant effect on the answerability and popularity of posts.

As for context variables, the *Topic diversity* variable was negatively related to comment count (*estimate*=−0.2139; *P*<.001) and positively associated with favorite count (*estimate*=0.6167; *P*<.001). The *Topic intensity* variable is positively related to comment count (*estimate*=−0.2010; *P*=.002), but the *P* value of *Topic intensity* variable is greater than .05, in terms of post popularity. These results show that posts with more topics may be difficult to answer but useful, and those with a larger degree of semantic network are more accessible to answers.

Regarding user-related variables, the *P* value of *Participation intensity* is less than .001 in *Model 2* and greater than .05 in *Model 4*. The *Sociability breadth* variables were significant in both *Model* *2* and *Model* *4*, with estimated values of 0.3016 and −0.5515, respectively. These results indicate that *Sociability breadth* is positively related to comment counts and negatively associated with favorite counts.

**Table 3:** Results of negative binomial regression model (answerability).

Variables		Model 1			Model 2	
		Estimate (SE)	*P* value	Estimate (SE)		*P* value
**Control variables**						
	Work time	−0.0045 (0.0080)	.58	−0.0076 (0.0080)		.34
	Content length	1.6977 (0.0000)	<.001	1.8482 (0.0658)		<.001
	Title length	−0.4728 (0.0002)	.05	−0.3813 (0.0513)		<.001
**Content variables**						
	Content readability	—^a^	—	0.1718 (0.0102)		<.001
	Title sentiment	—	—	−0.0837 (0.0169)		<.001
	|Title sentiment|	—	—	0.1070 (0.0190)		<.001
	Content sentiment	—	—	−0.2093 (0.0213)		<.001
	|Content sentiment|	—	—	0.0984 (0.0263)		<.001
**Context variables**						
	Topic diversity	—	—	−0.2139 (0.0563)		<.001
	Topic intensity	—	—	0.2010 (0.0655)		.002
**User variables**						
	Participation intensity	—	—	−0.0406 (0.0163)		.01
	Sociability breadth	—	—	0.3016 (0.0656)		<.001
**Model fit**						
	AIC^b^	203,114.15	—	202,501.86		—

^a^Variable has not been input.

^b^AIC: Akaike information criterion.

**Table 4:** Results of negative binomial regression model (Popularity).

Variables		Model 3			Model 4	
		Estimate (SE)	*P* value	Estimate (SE)		*P* value
**Control variables**						
	Work time	−0.0121 (0.0238)	.50	−0.0121 (0.0177)		.50
	Content length	2.7308 (0.1466)	<.001	2.8067 (0.1521)		<.001
	Title length	−0.2432 (0.1109)	.03	−0.4193 (0.1096)		<.001
**Content variables**						
	Content readability	—^a^	—	−0.2568 (0.0223)		<.001
	Title sentiment	—	—	0.3377 (0.0346)		<.001
	|Title sentiment|	—	—	0.2638 (0.0614)		<.001
	Content sentiment	—	—	0.2060 (0.0487)		<.001
	|Content sentiment|	—	—	0.3446 (0.0594)		<.001
**Context variables**						
	Topic diversity	—	—	0.6167 (0.1266)		<.001
	Topic intensity	—	—	0.1160 (0.1484)		.43
**User variables**						
	Participation intensity	—	—	−0.0408 (0.0370)		.27
	Sociability breadth	—	—	−0.5515 (0.1480)		<.001
**Model fit**						
	AIC^b^	151,890.91	—	151,543.71		—

^a^Variable has not been input.

^b^AIC: Akaike information criterion.

## Discussion

### Principal Findings

Although several studies have analyzed the factors influencing the answerability and popularity of posts in terms of content, user, and context [5,6,31,32], few have considered the sentiments of content-related features and the dynamics of user-related and context-related features. The experimental results in Tables 3 and 4 demonstrate a significant influence of sentiment tendency on the answerability and popularity of posts. Specifically, sentiment features have a negative influence on answerability and a positive influence on popularity. Wang et al [31] also found that positive feedback is negatively correlated with continuous answerability. Moreover, the absolute values of the title and content sentiment variables both have positive effects on the answerability and popularity of posts. The above results suggest that health users could appropriately convey sentiment trends, especially negative sentiments such as worry and anxiety, to gain more attention during health consultations.

With the dynamic evolution of social and semantic networks, the user and tag centrality values are also changing. The degree of centrality was dynamically calculated by constructing a temporal network. In this study, the users’ degree of centrality had more significant positive effects on the comment count in the following network. Qian et al [18] found that users’ centrality has a positive influence on the number of replies but ignores the dynamics of the user Q&A network. Accordingly, the centrality of tags (*Topic intensity*) has a significant influence only on the answerability of posts. These findings demonstrate that posts with common tags were more likely to be answered by other users. The tag count of a post has a negative influence on its answerability and positive effect on its popularity. Similarly, Shi et al [4] demonstrated that tag frequency is positively correlated with the popularity of posts.

Therefore, the main findings and results of this study are as follows. (1) Content-related variables have a strong impact on both the answerability and popularity of posts, with the readability and sentiment variables of the post being crucial factors. (2) User-related variables significantly affect the answerability and popularity of posts, and the degree of centrality of users in the social network serves as a key indicator. (3) Context-related variables have a more substantial influence on the popularity of posts than their answerability, with the topic diversity of the posts being an essential factor from the perspective of the estimate value and significance.

### Research and Practical Implications

In terms of research implications, this study provides valuable contributions to relevant theories. On the one hand, it examines the impact of user-related and context-related variables on consultation behavior from the perspective of the user following the dynamic network and tag semantic dynamic network; in contrast, from the standpoint of sentiment analysis, this study investigates the impact of sentiment inclination on answerability and popularity, demonstrating the role of sentiment variables in eliciting emotional resonance and fostering knowledge exchange. Overall, this study analyzed the factors influencing the answerability and popularity of posts simultaneously, which helps to contrast and reveal the different expectations of users regarding commenting and favoring. These findings would contribute to further explaining the underlying behavioral patterns of users in health Q&A communities.

As for practical implications, building on these findings, this research offers practical insights for users, platform operators, and health consultants within web-based Q&A communities. Specifically, this study revealed effective strategies to increase the answerability of posts, such as improving the readability and sentiment inclination of posts and labeling an appropriate number of tags. For platform operators, this study provides a theoretical basis for optimizing platform management, including encouraging users to use the tagging system and promoting social interactions among users. For users, this research serves as a reference guide to enhance consultation efficiency and quality, focusing on the readability and sentiment inclination of posts and recommending posts with strong topic intensity for answering.

### Limitations and Future Directions

Despite its contributions, this study had several limitations. First, it depends exclusively on data from a single web-based *question-and-answer* community, potentially leading to a sample bias. Subsequent studies could broaden the data sources and compare user consultation behaviors across various web-based Q&A communities. Second, this study considered only the answerability and popularity of posts as effectiveness indicators for consultation behavior. Further investigations could incorporate additional measures such as post quality, answer quality, and user satisfaction. In addition, this study focuses on the perspective of individuals posing posts; future research could examine the respondents’ viewpoints to explore the motivations and factors driving their involvement in consultation behavior.

### Conclusions

This study delved into the diverse factors that shape user health consultation behavior within web-based Q&A communities, encompassing content-related, user-related, and context-related variables. By collecting comprehensive user behavior data from the *Metafilter* *Q&A* community, we constructed dynamic social networks of users and dynamic semantic networks of tags and calculated indicators such as *participation intensity*, *sociability breadth*, *topic intensity*, etc. Altogether, different variables within the same model exhibited different levels of significance and impact intensity. The same variables had different effects on the answerability and popularity of posts.

## Appendix

Multimedia Appendix 1 Data set description.

## Abbreviations

NBRM: negative binomial regression model
OHQP: online health question-and-answer platform
Q&A: question-and-answer
VIF: variance inflation factor

## Data Availability

The data sets generated and analyzed during this study are available in the (figshare) repository [33].
