# Supplementary material for: Factors Influencing the Answerability and Popularity of a Health-Related Post in the Question-and-Answer Community: Infodemiology Study of Metafilter
Source: J Med Internet Res. 2023 Nov 17;25:e48858. doi: 10.2196/48858 (PMC10692880; doi:10.2196/48858)
Supplement: Multimedia Appendix 1 [file jmir_v25i1e48858_app1.docx]

**Appendix:**

The dataset utilized in this study was culled from the *Metafilter* database (*http://stuff.metafilter.com/infodump/*). *MetaFilter* is a general-interest online community that is composed of *MetaFilter* itself, *AskMeFi, FanFare, Projects, Music, Jobs, IRL*, and *MetaTalk* subsites, as shown in Figure A-1.


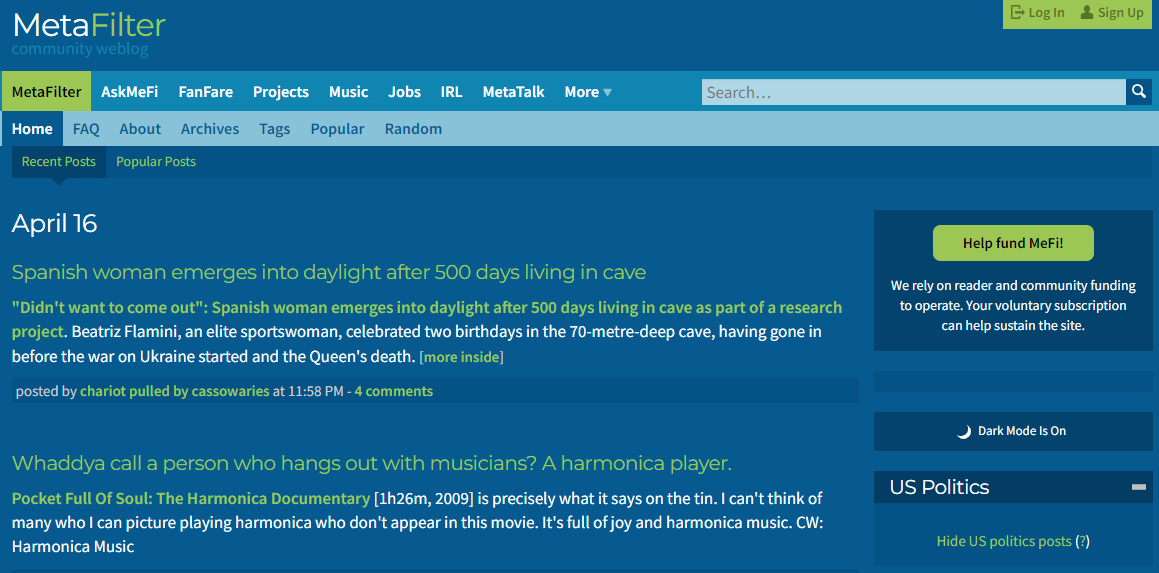


A-1. The home page of *MetaFilter* online community

Among them, *AskMeFi* is a popular question-and-answer (Q&A) community where users post a wide range of questions, seeking answers from the collective experience and knowledge of others. By December 2022, *AskMeFi* had 356,635 posts and 4,934,541 answers, with the topics of these posts spanning 20 categories, such as *clothing*, *beauty & fashion*, *computers & internet*, *education*, *food & drink*, *grab bag*, *health & fitness*, and more. And all posts are mostly about health consultation in the health & fitness category. The *AskMefi* archives category and health & fitness posts are shown in Figure. A-2.

Figure A-2. The screenshot of the *AskMefi* archives category and health & fitness posts

One of the major reasons for selecting the *AskMeFi* Q&A community is the availability of comprehensive user behavior data via open a download link (*http://stuff.metafilter.com/infodump/*). Besides, *AskMeFi* is one of seven subsites, the user behavior data in other sub sites also can be used for analyzing user health consultation behavior. Specifically, using the complete set of user activity data from the *MetaFilter* online community, we can construct both a dynamic social network of users and a dynamic semantic network of topics. The data structure diagram of *MetaFilter* is depicted in Figure A-3.


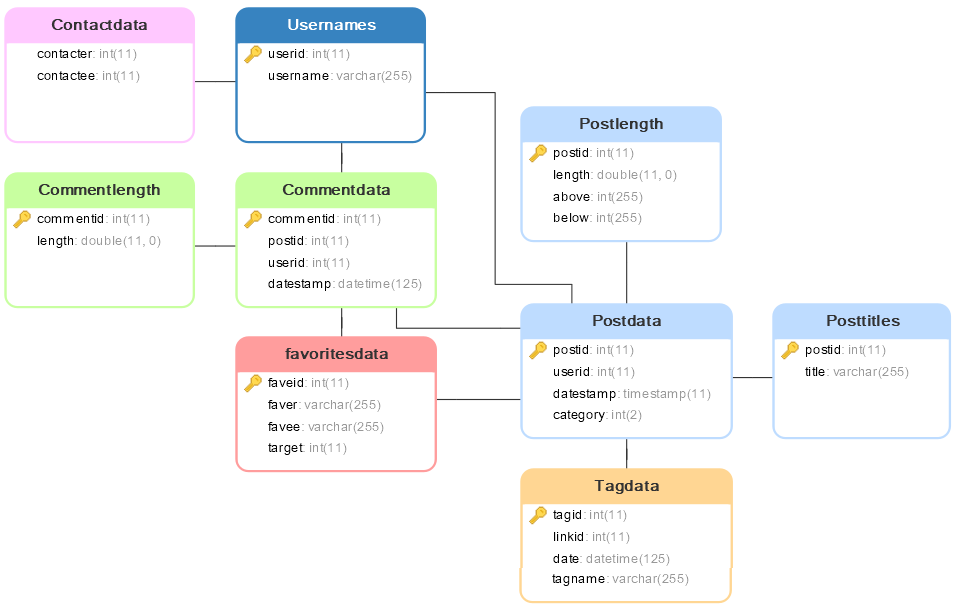


Figure A-3. The data structure diagram of *MetaFilter*

In Figure A-3, we can find that each sub site has comments, posts, and tags to record the process of user health consultation. The contact data records the history of one user linking to others, total number of contact records is 133,322 by December 2022. The favorite table has recorded 2,179,735 favorited posts and 37,373,216 favorited comments. The usernames table contains 75, 724 users, including userid and username items. We conducted time series statistics on comments and posts of *MetaFilter, AskMeFi* and *FanFare*, with the results displayed in Figure A-4.


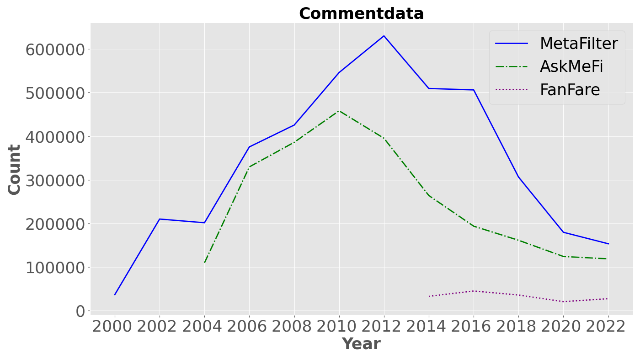

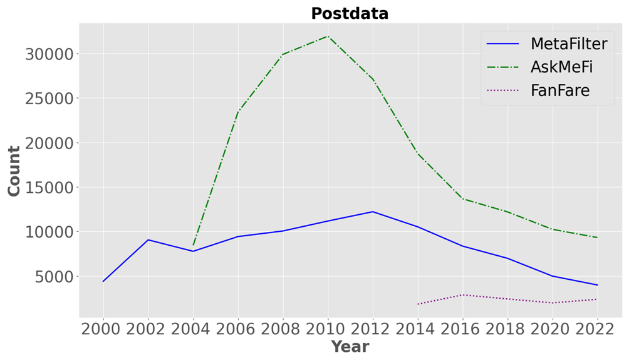


Figure A-4. The time series statistics about comments and posts of *MetaFilter, AskMeFi* *and FanFare*
